# A Planar-Dimensions Machine Vision Measurement Method Based on Lens Distortion Correction

**DOI:** 10.1155/2013/963621

**Published:** 2013-10-27

**Authors:** Qiucheng Sun, Yueqian Hou, Qingchang Tan, Guannan Li

**Affiliations:** ^1^College of Basic Science, Changchun University of Technology, Yanan Street 2055, Changchun 130012, China; ^2^College of Mechanical Science and Engineering, Changchun University, Changchun 130012, China; ^3^College of Mechanical Science and Engineering, Jilin University, Changchun 130012, China

## Abstract

Lens distortion practically presents in a real optical imaging system causing nonuniform geometric distortion in the images and gives rise to additional errors in the vision measurement. In this paper, a planar-dimensions vision measurement method is proposed by improving camera calibration, in which the lens distortion is corrected on the pixel plane of image. The method can be divided into three steps: firstly, the feature points, only in the small central region of the image, are used to get a more accurate perspective projection model; secondly, rather than defining a uniform model, the smoothing spline function is used to describe the lens distortion in the measurement region of image, and two correction functions can be obtained by fitting two deviation surfaces; finally, a measurement method for planar dimensions is proposed, in which accurate magnification factor of imaging system can be obtained by using the correction functions. The effectiveness of the method is demonstrated by applying the proposed method to the test of measuring shaft diameter. Experimental data prove that the accurate planar-dimensions measurements can be performed using the proposed method even if images are deformed by lens distortion.

## 1. Introduction

Image measurement has advantages of noncontact, fast speed, and high precision and has applications in industry, medicine, and other fields [1–3]. Because most mechanical part sizes are often in plane, planar-dimensions measurement (2D measurement) has been widely used in the field of industrial measurement [4, 5]. This has several advantages compared to a 3D vision measurement, such as, for example, (a) reduction in costs (only one camera involved) and (b) more precise (no cross-camera matching and triangulation). Since no triangulation procedures are involved in 2D measurements, the camera calibration is often omitted and lens distortions are also not corrected [6].

In fact, for real camera lenses, such as a fixed length lens, a zoom lens, or even an expensive high-quality telecentric lens, image distortions unavoidably exist due to lens aberrations and misalignment of optical elements [7]. Due to the nonuniform characteristics of lens distortion, the imaging of mechanical parts in the sensor plane may be warped. Clearly, these distortions in the images caused by lens distortion can be detected by the subpixel detection algorithms and hence corrupt the real size of mechanical parts, reducing the accuracy of 2D measurements. With the precision requirement of measurement becoming higher, the errors due to lens distortion are much worth to be considered and should therefore be eliminated. Though various powerful camera calibration techniques [8–10] have been developed in the field of computer vision and have also been successfully used in 3D measurement, these techniques seem to be too complicated for 2D measurement, which corrects the lens distortion on a hypothetical image plane in calibration model rather than pixel plane of real image [11]. A simple, easy-to-implement yet effective lens distortion correction method is therefore necessary.

In practice, lens distortion can be regarded as a system error since camera and lens are fixed. Therefore, it is not necessary that uniform model of image distortions is used for the camera calibration [12, 13]. Since the influence of image distortion is related to position with respect to principal point (closing to image center), the center region of image rather than the whole image is used generally for the measurement to ensure accuracy. So, in the present work, a planar-dimensions vision measurement method is proposed by correcting lens distortion in a region of interest (measurement region) on the pixel plane. Firstly, a linear camera model and the feature points near the image center are used to calibrate the perspective projection; then, the image distortion in the measurement region, considering the region larger than the center area, is corrected using the smoothing spline function; finally, a method of measuring planar dimensions is proposed by means of the function. Accuracy and performance of the method are tested by a measurement experiment. 

The organization of the paper is as follows: Section 2 presents a linear calibration model. The distortion correction function is proposed in Section 3. A planar-dimensions vision measurement method is given and an experiment of measuring shaft diameter is implemented in Section 4. Finally, a conclusion is made in Section 5.

## 2. The Calibration of Perspective Projection

At present, the pinhole projective model was calibrated by mapping 3D scenes to the 2D camera image plane in the literature [10]. The mapping from the world points to the image ones can be expressed as follows:

(1) $$\begin{array}{c} s\left[\begin{array}{c} x_{u}\\ y_{u}\\ 1 \end{array}\right]=\left[RT\right]\left[\begin{array}{c} X_{w}\\ Y_{w}\\ Z_{w}\\ 1 \end{array}\right], \end{array}$$

(2) $$\begin{array}{c} \left[\begin{array}{c} x_{d}\\ y_{d} \end{array}\right]=\left(1+k_{1}r^{2}+k_{2}r^{4}\right)\left[\begin{array}{c} x_{u}\\ y_{u} \end{array}\right], \end{array}$$

(3) $$\begin{array}{c} \left[\begin{array}{c} u_{p}\\ v_{p}\\ 1 \end{array}\right]\left[\begin{array}{ccc} \alpha & \gamma & C_{x}\\ 0 & \beta & C_{y}\\ 0 & 0 & 1 \end{array}\right]\left[\begin{array}{c} x_{d}\\ y_{d}\\ 1 \end{array}\right], \end{array}$$

where $r=\sqrt{x_{u}^{2}+y_{u}^{2}}$. And the calibration is finished using the Levenberg-Marquardt algorithm. Here, (2), whose form is determined, corrects radial distortion of a camera. However, in real imaging system, the distribution of distortion in the image is not uniform, and the distortion near the projection center is very small based on past experience. Therefore, it is not necessary that (2) is used to correct the lens distortion in the centre area. So, this work puts forward a method of calibrating perspective projection accurately. In the method, a pinhole model and the feature points near the projection center can be used to calibrate a camera without considering image distortion. In this way, a linear calibration model can be obtained by using (1), (3) and ignoring (2):

(4) $$\begin{array}{c} s\left[\begin{array}{c} u_{p}\\ v_{p}\\ 1 \end{array}\right]=A\left[\begin{array}{ccc} r_{1} & r_{2} & t \end{array}\right]\left[\begin{array}{c} X_{w}\\ Y_{w}\\ 1 \end{array}\right]=H\left[\begin{array}{c} X_{w}\\ Y_{w}\\ 1 \end{array}\right], \end{array}$$

*Z*_*w*_ = 0 in the model, where *r*_*i*_  (*i* = 1,2) is the *i*th column vector of the rotation matrix, *t* is the translation vector, $A=\left[\begin{array}{ccc} \alpha & \gamma & u_{0}\\ 0 & \beta & v_{0}\\ 0 & 0 & 1 \end{array}\right]$ is intrinsic parameters matrix, and the homography *H* is a 3 × 3 matrix. 

**Figure 1:**
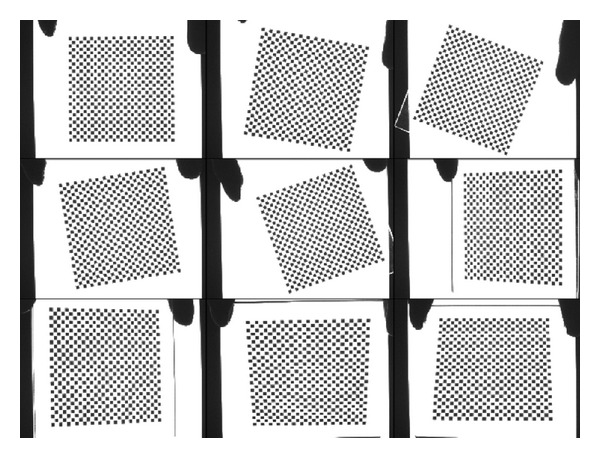
Patterns for the calibration.

**Figure 2:**
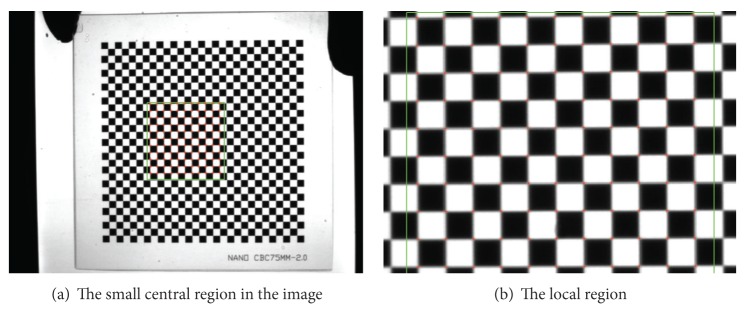
The feather points in the central region of 300 × 300 pixels.

In the experiment, nine patterns of a check board are acquired by the camera with a 25 mm fixed lens, and the image resolution is 1376 × 1024 pixels, as shown in Figure 1. Size of grids on the board is 2 × 2 mm, and corner points of the patterns are detected using the method in reference [14]. In this paper, only the corner points in 300 × 300 pixels region around the projection center of patterns are used in (4) to calibrate the camera, as shown in Figure 2. The residual of calibration on the world coordinate can be calculated by the following formula:

(5) $$\begin{array}{c} E_{i}=\frac{1}{n}\overset{n}{\underset{j=1}{\sum }}{||M-{\overset{⌢}{M}}_{ij}||}_{2},\;i=1,\ldots ,9, \end{array}$$

where *E*_*i*_ is the mean residual of the *i*th pattern in metric units, *n* is number of the corner points used on one pattern, *M* denotes the world coordinate of the corner points, and $\overset{⌢}{M}$ does the world coordinate of the corner points calculated by the model (4). By this way, the residuals of corner points only in the 300 × 300 region of each pattern are calculated and are listed in Table 1. In order to compare, Table 1 also gives the residuals of the central region calculated by means of Zhang's model, which is calibrated using all the corner points of the patterns.

Data of Table 1 show that the present method can achieve high calibration accuracy in the central region of 300 × 300 pixels. This is since the distortion of the points near the image center is very small, and the accurate perspective projection can be obtained. Because Zhang's model is influenced greatly by the points away from the center region, calibration accuracy of the central region will eventually be sacrificed.

## 3. Smoothing Spline Distortion Model

**Figure 3:**
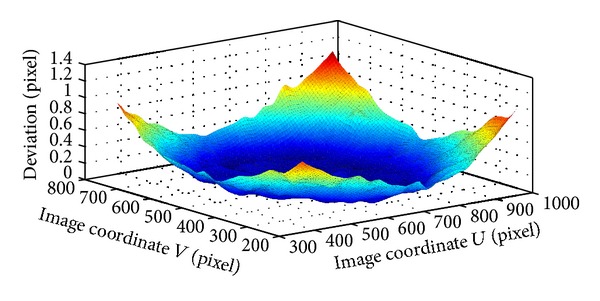
Deviation in 600 × 600 pixels.

In practice, the image region used for the measurement is usually larger than that for the calibration. This may affect the measurement accuracy since the imaging system is not perfect, such as the lens distortion. Now, deviation between corner positions on the board pattern and ones calculated by (4) is considered, as shown in Figure 3. The deviation values for the region between 600 × 600 pixels and 300 × 300 ones are large since the homography matrix *H* in (4) is calibrated by points in region of 300 × 300 pixels. So, the predication, which is given by (4) in the region of 300 × 300 pixels outside, is corrected by the check board. And the correction can be expressed by

(6) $$\begin{array}{c} u_{p}=u_{d}+\delta_{u}\left(u_{d},v_{d}\right),\\ v_{p}=v_{d}+\delta_{v}\left(u_{d},v_{d}\right), \end{array}$$

where *u*_*p*_ and *v*_*p*_ are the undistortion image coordinates on the pixel plane projected from (4), *u*_*d*_ and *v*_*d*_ are the distortion image coordinates extracted from the board patterns, and *δ*_*u*_ and *δ*_*v*_ are deviations of the points on the coordinate axis *U* and *V*. And correction of (6) can be conveniently governed as a surface fitting problem:

(7) $$\begin{array}{c} \delta_{u}=f_{u}\left(u_{d},v_{d}\right)\\ \delta_{v}=f_{v}\left(u_{d},v_{d}\right). \end{array}$$

Regarding the correction, the previous papers [8–10] used a uniform mathematical model to describe the radial, decentering, and prism distortions in whole image. However, the distortions are related to a specific imaging system and cannot be represented by the uniform model [12, 13]. Therefore, (7) use a union of spline function to describe the distortion only in the local region, and this surface fitting of (7) is finished by the smoothing spline algorithm [15, 16].

**Figure 4:**
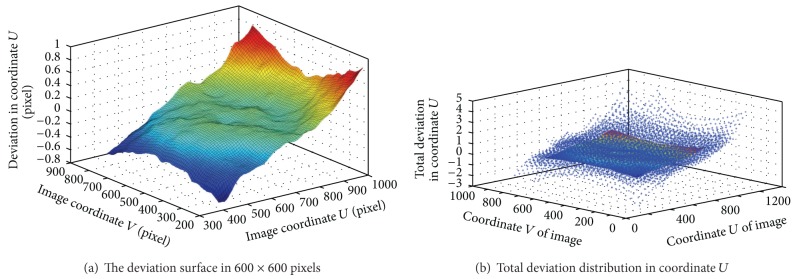
Deviation surface in coordinate *U* of the image.

**Figure 5:**
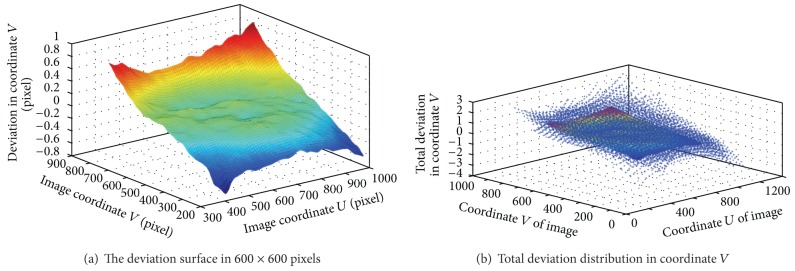
Deviation surface in coordinate *V* of the image.

**Figure 6:**
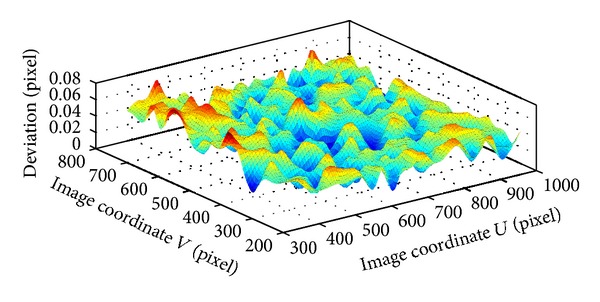
Deviation surface after correction.

As an example, the deviation in center region of 600 × 600 pixels is corrected based on (7) by smoothing spline algorithm. Since the homography matrix *H*_*i*_ have been calibrated based on (4) by using the points in area of 300 × 300 pixels, the world coordinates of corner points in nine patterns can be projected onto the pixel coordinates. By this way, the total deviation distribution in the *U* and *V* axis of the image is acquired by (6). Using the smoothing spline algorithm, two distortion correction functions can be obtained, and two deviation surfaces are shown in Figures 4 and 5. It can be observed in the Figure 6 that the deviation is very small after correction. 

## 4. Planar-Dimensions Vision Measurement Method

A planar-dimensions measurement method is proposed by means of the distortion correction functions and used to measure the shaft diameter as an example in this section. Although the shaft is a 3D object, the measurement of shaft diameter can be considered as a 2D measurement when the optical axis of the imaging lens is perpendicular to center line of shaft approximatively.

**Figure 7:**
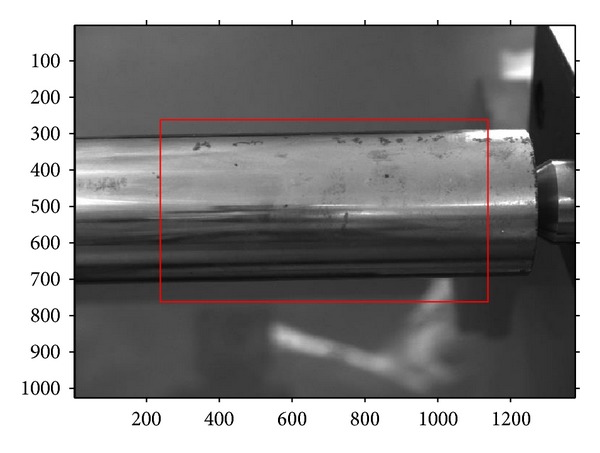
The measurement region of shaft diameter.

It can be seen in Figure 7 that the central region of 900 × 500 pixels contains the main portion of the shaft, whose diameter is about 40 mm. That is to say, this region can be used as a measurement region according to this lens, and the measurement range is supposed to be about 40 mm. Then, the measurement is carried by the following steps:

1. using the proposed calibration method, the central image region of 900 × 500 pixels can be calibrated and two distortion correction functions can be obtained in the *U* and *V* axis of the image;
2. two edges of the shaft are detected using sub-pixel edge detection method [17–19], and the pixel coordinates of edge points are corrected by the distortion correction functions;
3. two parallel lines are fitted using the corrected points in the plane of pixel, and pixel distance between the two lines can be obtained.

**Figure 8:**
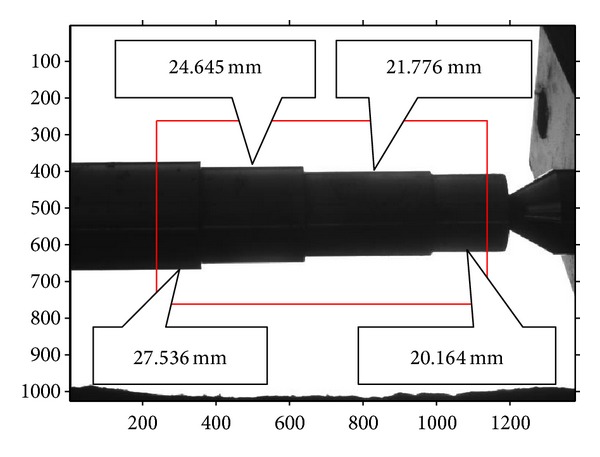
The imaging of four-segment shaft.

**Figure 9:**
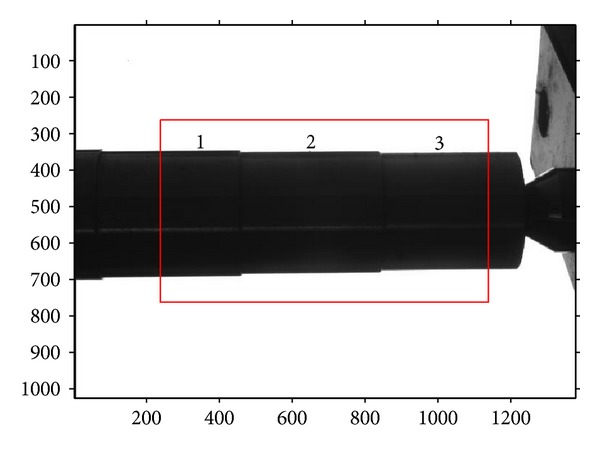
The imaging of three-segment shaft.

**Figure 10:**
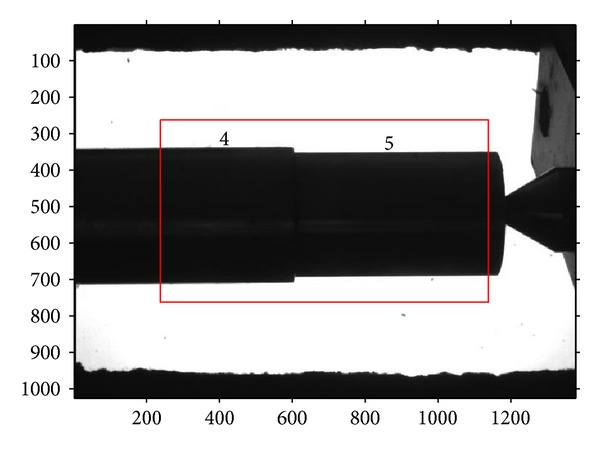
The imaging of two-segment shaft.

**Figure 11:**
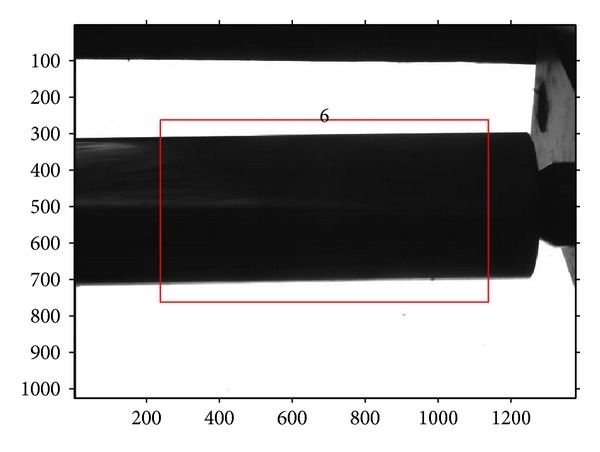
The imaging of one-segment shaft.

A four-segment shaft, whose diameters are known, should be measured using the above measurement procedures firstly, as shown in Figure 8. In this way, the metric length per pixel in the measurement region can be obtained. Then, the other shafts, shown in Figures 9, 10, and 11, can be measured through the same operation with a known per-pixel, and the measured values are listed in Table 2. In order to compare them, Table 2 also gives the values measured using edge points without correction. Through comparison with real diameters (measured by Electronic Digital Outside Micrometer), the mean absolute error 0.0045 mm and variance 3.1 × 10^−6^ of the proposed method can be calculated, which are smaller than the measurements without correction. So, the proposed method can improve the 2D measurement accuracy efficiently.

## 5. Conclusion

This study develops a machine vision method for high-precision 2D measurement. In the method, a novel algorithm is proposed by improving the calibration model. In this way, the lens distortion can be corrected on the pixel plane before measuring, and accurate magnification factor of imaging system can be obtained. Experimental results indicate that the proposed method possesses a precision of 0.005 mm for measuring shaft diameter about 40 mm.

## Figures and Tables

**Table 1:** The residuals of the proposed and Zhang's methods in the central region (*μ*m).

Patterns	1	2	3	4	5	6	7	8	9
Proposed method	3.0	4.1	4.8	4.4	4.6	3.8	3.6	3.3	3.3
Zhang's method	5.3	5.8	6.1	5.9	6.0	5.6	5.5	5.4	5.4

**Table 2:** Measurement results of segments of shaft (mm).

Segment of shaft	1	2	3	4	5	6
Known values	32.441	31.467	30.459	34.422	31.589	37.725
Proposed method	32.447	31.469	30.463	34.426	31.593	37.732
Without correction	32.506	31.526	30.520	34.501	31.650	37.827
